# Synthesis of new pyridothienopyrimidinone derivatives as Pim-1 inhibitors

**DOI:** 10.1080/14756366.2016.1261130

**Published:** 2017-01-18

**Authors:** Bassem H. Naguib, Hala B. El-Nassan, Tamer M. Abdelghany

**Affiliations:** a Pharmaceutical Chemistry Department, Faculty of Pharmacy, The British University in Egypt, Cairo, Egypt;; b Pharmaceutical Organic Chemistry Department, Faculty of Pharmacy, Cairo University, Cairo, Egypt;; c Department of Pharmacology and Toxicology, Faculty of Pharmacy, Al-Azhar University, Cairo, Egypt

**Keywords:** Pyridothienopyrimidine, pyrido[3',2':4,5]thieno[3,2-d]pyrimidin-4(3H)-one, cytotoxic activity, pim-1 inhibitors

## Abstract

Four series of pyridothienopyrimidin-4-one derivatives were designed and prepared to improve the pim-1 inhibitory activity of the previously reported thieno[2,3-*b*]pyridines. Significant improvement in the pim-1 inhibition and cytotoxic activity was achieved using structure rigidification strategy *via* ring closure. Six compounds (**6c**, **7a, 7c**, **7d**, **8b** and **9**) showed highly potent pim-1 inhibitory activity with IC_50_ of 4.62, 1.18, 1.38, 1.97, 8.83 and 4.18 μM, respectively. Four other compounds (**6b**, **6d**, **7b** and **8a**) showed moderate pim-1 inhibition. The most active compounds were tested for their cytotoxic activity on three cell lines [MCF7, HCT116 and PC3]. Compounds **7a** [the 2-(2-chlorophenyl)-2,3-dihydro derivative] and **7d** [the 2-(2-(trifluoromethyl)-phenyl)-2,3-dihydro derivative] displayed the most potent cytotoxic effect on the three cell lines tested consistent with their highest estimated pim-1 IC_50_ values.

## Introduction

The Provirus Integration in Maloney (Pim) kinases represent a family of constitutively active serine/threonine kinases and include three subtypes (pim-1, pim-2 and pim-3). Pim kinases regulate many biological processes such as cell cycle, cell proliferation, apoptosis and drug resistance^1–4^. Being expressed in many types of solid and hematological cancers and almost absent in benign lesions, pim kinases proved to be a successful anti-cancer drug target of low toxicity^5–12^.

Most of the research published on pim inhibitors focused on pim-1 inhibitors while pim-2 is more difficult to be targeted due to its low K_m_ for ATP (100-fold lower than that of Pim-1 and Pim-3)^13–16^.

Recently reported manuscripts indicated that pim-1 kinase plays a significant role in stem cell proliferation, self-renewal and expansion^17^. These facts encourage the use Pim-1 inhibitors as a promising targeted therapy in cancer stem cells^5^^,^^17^.

**Figure 1.**
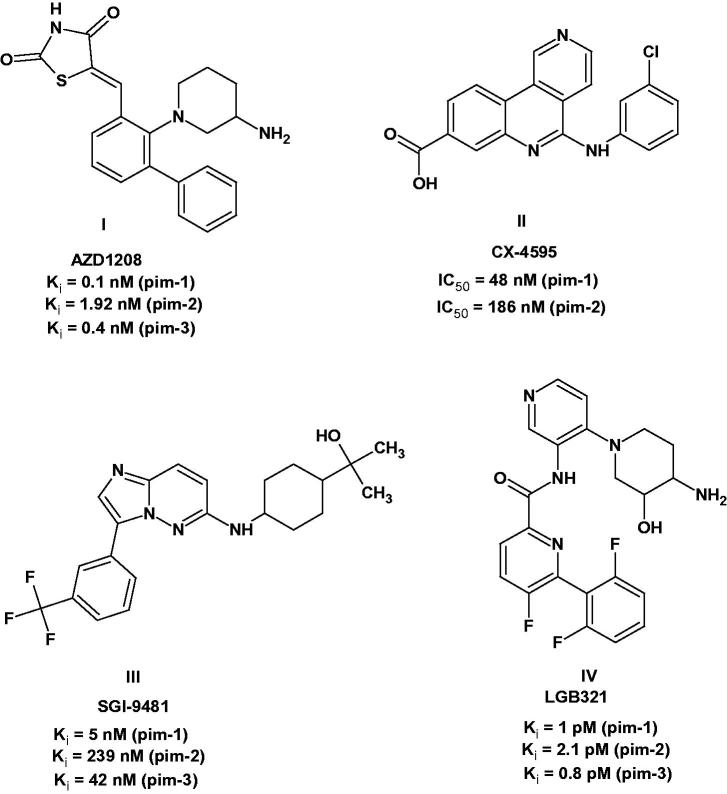
Pim-1 inhibitors under clinical and preclinical studies.

Indeed, some of the pim-1 inhibitors have entered phase I clinical trials such as the thiazolidin-2,4-dione derivative AZD1208 (**I**)^1^^,^^18^ and the benzonaphthyridine derivative CX-4595 (**II**)^1^^,^^19^^,^^20^. While others such as the imidazo[1,2-*b*]pyridazine derivative SGI-9481 (also known as TP-3654, **III**)^1^ and the highly potent pyridine derivative LGB321 (**IV**)^1^^,^^21^^,^^22^ are under preclinical studies (Figure 1).

The most important feature of pim-1 kinase active site is the presence of proline base at positions 123 and 125 within the hinge region. This extends the hinge region length and moves it 4 A^o^ to the left and thus prevents the formation of the second H-bond between the hinge backbone and the adenine moiety of ATP^23^.

Almost all the reported pim kinase inhibitors are ATP-competitive and can be classified into ATP-mimetics and non-ATP mimetics. The ATP-mimetics bind directly to the hinge region usually *via* H-bonding with the backbone oxygen of Glu121 and exhibited great enzyme potency but with limited or poor selectivity over other kinases^1^. On the other hand, the non-ATP mimetics bind to the ATP active site in a manner different from ATP and most of them form H-bond with Lys67^1^. Generally, they interact with the portion of the active site opposite to the hinge region and this portion differs significantly between kinases. Thus, non-ATP mimietics tend to be more selective to pim-1 enzyme and meanwhile exhibited great potency to the enzyme. Compounds **I**–**IV** (Figure 1) are all non-ATP mimietics.

**Figure 2.**
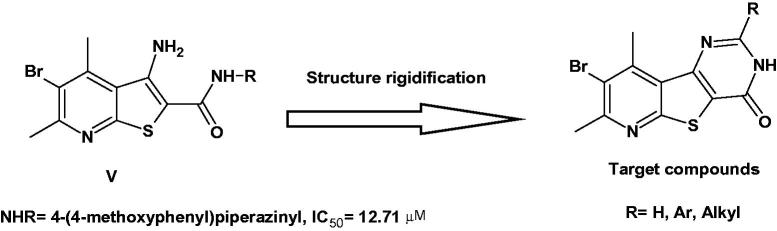
Designing pyridothienopyrimidinones as Pim-1 inhibitors.

In an attempt to prepare potent pim-1 inhibitors that can be used as anticancer agents, we had recently reported the pim-1 inhibitory activity of thieno[2,3-*b*]pyridine derivatives **V** (Figure 2)^24^ as bioisosteres to benzofuran-2-carboxylic acids^25^. However, their pim-1 inhibitions as well as their cytotoxic activities were only moderate to poor. This literature recorded some of the efforts done by our lab to improve the pim-1 kinase inhibitory activity and the cytotoxic activity of thieno[2,3-*b*]pyridine scaffold.

Upon designing thieno[2,3-*b*]pyridine derivatives as isosteres to benzofuran-2-carboxylic acids, we assumed that the amidic C=O can form H-bond with Lys67. Therefore, the poor activity achieved by these derivatives might originate from the flexibility of the amidic bond resulting in improper orientation of the carbonyl group towards Lys67. Accordingly, structure rigidification *via* ring closure of the 3-amino-thieno[2,3-*b*]pyridine-2-carboxamide core into pyrido[3',2':4,5]thieno[3,2-*d*]pyrimidin-4(3*H*)-one scaffold might orient the carbonyl group properly towards Lys67.

**Figure 3.**
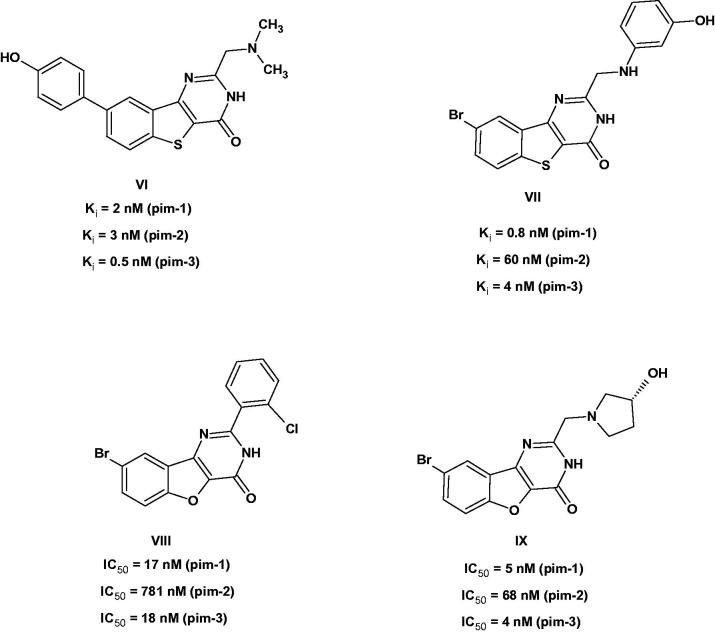
Examples of benzothienopyrimidinones and benzofuropyrimidinones as Pim-1 inhibitors.

Searching the literature revealed no published data on pyridothienopyrimidinones as pim-1 kinase inhibitors. However, their bioisosteres benzothienopyrimidin-4-ones **VI** and **VII**^14^^,^^26^^,^^27^ and benzofuropyrimidin-4-ones **VIII** and **IX**^28^^,^^29^ had been reported earlier and exhibited potent and selective pim kinase inhibition (Figure 3). The X-ray crystallography of representatives of both series bound to pim-1 showed similar binding mode in the ATP-binding site^27^^,^^29^. In both series, the 4-carbonyl group formed H-bond to Lys67, while the group at the 8-position (Br or aryl) occupied the hydrophobic area against the hinge region. The substituent at position 2 was exposed to the solvent and thus can accommodate various groups, however, the best results were achieved with *ortho*-substituted phenyl or aliphatic amines like dimethylaminomethylene group^27^^,^^29^. As such, both series might act as non-ATP mimetics.

**Scheme 1.**
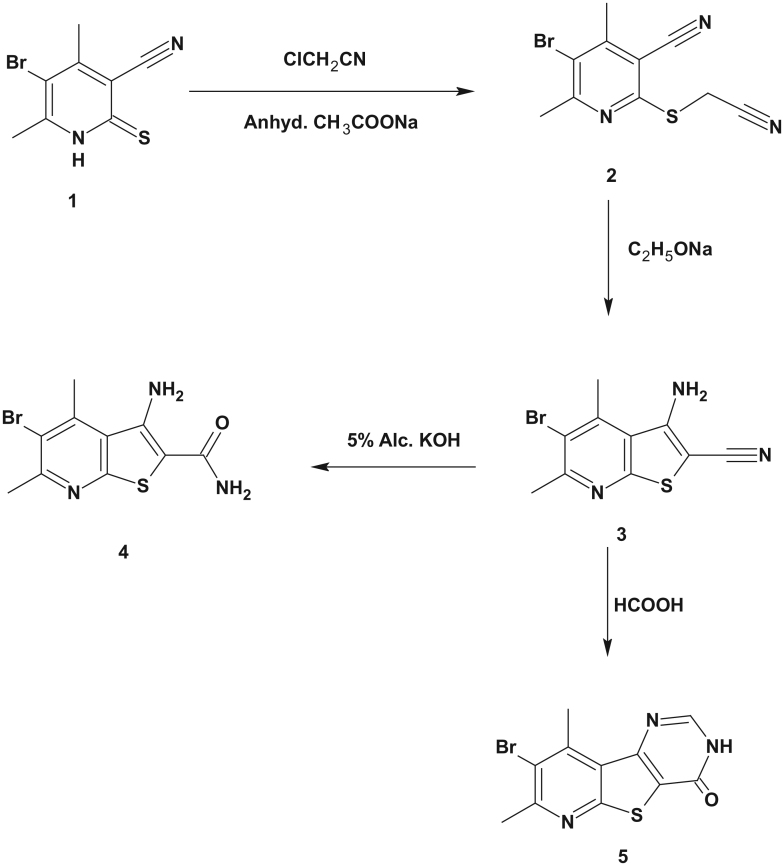
Synthesis of the starting compound**s 1–5**.

**Scheme 2.**
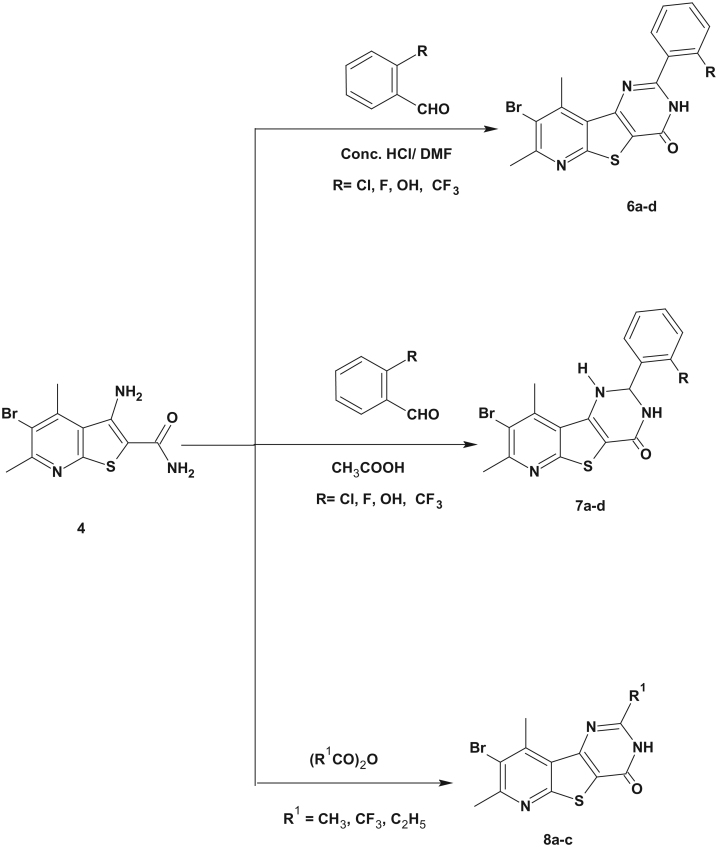
Synthesis of the target compounds **6–8**.

**Scheme 3.**
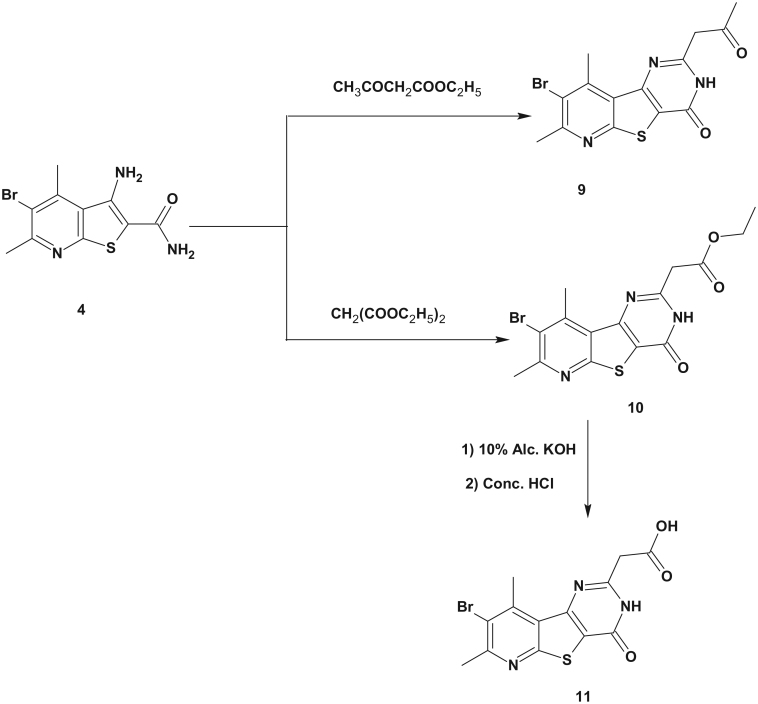
Synthesis of the target compounds **9–11**.

Encouraged by all the after mentioned findings, we thought of examining the pyridothienopyrimidinone scaffold as a novel class of pim-1 inhibitors. Here in, the bromine atom at position 8 was kept constant to ensure hydrophobic interaction within the pim-1 ATP active site. The carbonyl group at position 4 was also kept constant to ensure binding to Lys67. However, different substitutions were introduced at position 2 in order to establish a SAR for this new scaffold. First, the unsubstituted derivative **5** was prepared. Then, two series of 2-substituted phenyl compounds and two series of alkyl substituted derivatives were prepared. The *ortho*-substituted phenyl series (**6a**–**d**) was prepared to mimic compound **VIII**. The *ortho*-substitution used were chloro and flouro as halogens, OH as electron donating group and CF_3_ as bulky, hydrophobic and electron withdrawing group. In the second series (**7a**–**d**), the 2,3-dihydropyrimidinones, bearing the same 2-aryl groups as **6a**–**d**, were prepared to test whether aromaticity of the pyrimidinone ring was essential for enzyme activity or not. In the third series (**8a**–**c**), different alkyl groups with different chain lengths were prepared. In the last series (**9**–**11**), carbonyl containing alkyl groups were prepared (Schemes 1–3). Docking study of these compounds in the pim-1 active site indicated the required binding mode with high energy scores (data not shown).

All the synthesized compounds were examined for their pim-1 enzyme inhibitory activity and the most active compounds were further tested for their anti-proliferative activity using three different cell lines. Up to our knowledge, this is the first published work describing the pim-1 inhibitory activity of pyridothienopyrimidinone derivatives.

## Experimental

### General notes

Griffin apparatus was used to determine the melting points and they were uncorrected. Shimadzu IR 435 spectrophotometer recorded the IR spectra and the values were represented in cm^−1^. Bruker 400 MHz and 100 MHz spectrophotometer recorded ^1^H NMR and ^13^C NMR spectra, respectively. TMS was used as an internal standard and chemical shifts were recorded in ppm on δ scale. Both IR and NMR spectra were carried out at Faculty of Pharmacy, Cairo University, Cairo, Egypt. The electron impact (EI) mass spectra were recorded on Thermo Scientific ISQLT single quadrapole mass spectrometer. Both mass spectra and elemental analyses were carried out at the regional center for mycology and biotechnology, Al-Azhar University, Cairo, Egypt. Analytical thin layer chromatography (TLC) on silica gel plates containing UV indicator was employed routinely to follow the course of the reactions and to check the purity of the products. All reagents and solvents were purified and dried by standard techniques.

5-Bromo-4,6-dimethyl-2-thioxo-1,2-dihydropyridine-3-carbonitrile (**1**), 5-bromo-2-[(cyanomethyl)sulfanyl]-4,6-dimethylpyridine-3-carbonitrile (**2**), 3-amino-5-bromo-4,6-dimethylthieno[2,3-*b*]pyridine-2-carbonitrile (**3**) and 8-bromo-7,9-dimethylpyrido[3′,2′:4,5]thieno[3,2-*d*]pyrimidin-4(3*H*)-one (**5**) were prepared according to the published methods^30^.

### 3-Amino-5-bromo-4,6-dimethylthieno[2,3-*b*]pyridine-2-carboxamide (4)

A mixture of 3-amino-5-bromo-4,6-dimethylthieno[2,3-*b*]pyridine-2-carbonitrile (**3**) (1 g, 3.5 mmol) and 5% alc. KOH solution (1 g KOH in 20 mL ethanol) was heated under reflux for 3 h. The reaction was cooled and diluted with water (100 mL). The solid was filtered, dried and crystallized from ethanol.

Yield: 88%; mp: 260–261 °C^31^; IR (cm^−1^): 3464, 3417, 3317, 3267 (two NH_2_), 2924, 2854 (CH-aliphatic), 1666 (C = O); ^1^H NMR (DMSO-*d*_6_) δ ppm 2.68 (s, 3H, CH_3_), 2.86 (s, 3H, CH_3_), 6.89 (s, 2H, NH_2_, D_2_O exchangeable), 7.25 (s, 2H, NH_2_, D_2_O exchangeable); Anal. Calcd for C_10_H_10_BrN_3_OS: C, 40.01; H, 3.36; N, 14.00. Found: C, 40.23; H, 3.40; N, 14.21.

### General procedure for the synthesis of 8-bromo-2-(2-substituted phenyl)-7,9-dimethylpyrido[3',2':4,5]thieno[3,2-*d*]pyrimidin-4(1*H*)-ones 6a-d

A mixture of 3-aminothieno[2,3-*b*]pyridine-2-carboxamide **4** (0.6 g, 2 mmol), the appropriate 2-substituted benzaldehyde (2 mmol) and conc. HCl (0.5 mL) in DMF (5 mL) was heated under reflux for 12 h. The reaction was cooled, and the solid formed was filtered, dried and crystallized from the suitable solvent.

### 8-Bromo-2-(2-chlorophenyl)-7,9-dimethylpyrido[3',2':4,5]thieno[3,2-*d*]pyrimidin-4(3*H*)-one (6a)

Crystallized from DMF. Yield: 69%; mp: >300 °C; IR (cm^−1^): 3421 (NH), 2962, 2924 (CH-aliphatic), 1662 (C = O); ^1^H NMR (DMSO-*d*_6_) δ ppm 2.74 (s, 3H, CH_3_), 3.00 (s, 3H, CH_3_), 7.53–7.78 (m, 4H, Ar-H), 13.36 (s, 1H, NH, D_2_O exchangeable); ^13^C NMR (DMSO-*d*_6_) δ ppm 19.7, 26.8 (CH_3_), 122.9, 125.7, 127.8, 130.4, 131.8, 132.1, 132.5, 133.3, 144.2, 146.8, 151.6, 154.2, 158.4, 159.8 (aromatic carbons), 161.8 (C = O); Anal. Calcd for C_17_H_11_BrClN_3_OS: C, 48.53; H, 2.64; N, 9.99. Found: C, 48.70; H, 2.67; N, 10.14.

### 8-Bromo-2-(2-fluorophenyl)-7,9-dimethylpyrido[3',2':4,5]thieno[3,2-*d*]pyrimidin-4(3*H*)-one (6b)

Crystallized from DMF. Yield: 53%; mp: 295–296 °C; IR (cm^−1^): 3402, (NH), 2962, 2920 (CH-aliphatic), 1670 (C = O); ^1^H NMR (DMSO-*d*_6_) δ ppm 2.76 (s, 3H, CH_3_), 3.07 (s, 3H, CH_3_), 7.41–7.90 (m, 4H, Ar-H), 13.26 (s, 1H, NH, D_2_O exchangeable); ^13^C NMR (DMSO-*d*_6_) δ ppm 19.0, 23.1 (CH_3_), 117.5, 123.3, 128.8, 129.2, 129.9, 132.4, 134.4, 137.7, 140.2, 143.8, 154.8, 155.6, 156.6, 164.4 (aromatic carbons), 170.9 (C = O); MS m/z: 405 [(M + 2)^+^, 17.34%], 403 [M^+^, 21.19%], 43 [100%]; Anal. Calcd for C_17_H_11_BrFN_3_OS: C, 50.51; H, 2.74; N, 10.39. Found: C, 50.76; H, 2.81; N, 10.53.

### 8-Bromo-2-(2-hydroxyphenyl)-7,9-dimethylpyrido[3',2':4,5]thieno[3,2-*d*]pyrimidin-4(3*H*)-one (6c)

Crystallized from acetic acid. Yield: 57%; mp: 190–191 °C; IR (cm^−1^): 3394, 3309 (NH/OH), 2924, 2854 (CH-aliphatic), 1662 (C = O); ^1^H NMR (DMSO-*d*_6_) δ ppm 2.84 (s, 3H, CH_3_), 2.98 (s, 3H, CH_3_), 6.80–7.44 (m, 4H, Ar-H), 8.72 (s, 1H, OH, D_2_O exchangeable), 12.82 (s, 1H, NH, D_2_O exchangeable); Anal. Calcd for C_17_H_12_BrN_3_O_2_S: C, 50.76; H, 3.01; N, 10.45. Found: C, 50.98; H, 3.04; N, 10.67.

### 8-Bromo-7,9-dimethyl-2-[2-(trifluoromethyl)phenyl]pyrido[3',2':4,5]thieno[3,2-*d*]pyrimidin-4(3*H*)-one (6d)

Crystallized from DMF. Yield: 61%; mp: 255–256 °C; IR (cm^−1^): 3464 (NH), 2954, 2924 (CH-aliphatic), 1670 (C = O); ^1^H NMR (DMSO-*d*_6_) δ ppm 2.66 (s, 3H, CH_3_), 2.84 (s, 3H, CH_3_), 7.81–7.97 (m, 4H, Ar-H), 13.45 (s, 1H, NH, D_2_O exchangeable); ^13^C NMR (DMSO-*d*_6_) δ ppm 20.1, 26.8 (CH_3_), 121.1, 122.1, 125.0, 131.3, 132.8, 144.4, 146.6, 147.9, 151.2, 154.5, 156.9, 157.2, 158.3, 159.8, 162.7 (CF_3_ and aromatic carbons), 167.4 (C = O); MS m/z: 455 [(M + 2)^+^, 14.02%], 453 [M^+^, 17.20%], 77 [100%]; Anal. Calcd for C_18_H_11_BrF_3_N_3_OS: C, 47.59; H, 2.44; N, 9.25. Found: C, 47.67; H, 2.48; N, 9.44.

### General procedure for the synthesis of 8-bromo-2-(2-substituted phenyl)-7,9-dimethyl-2,3-dihydropyrido[3',2':4,5]thieno[3,2-*d*]pyrimidin-4(1*H*)-ones 7a–d

A mixture of 3-aminothieno[2,3-*b*]pyridine-2-carboxamide **4** (0.6 g, 2 mmol) and the appropriate 2-substituted benzaldehyde (2 mmol) in glacial acetic acid (5 mL) was heated under reflux for 10 h. The reaction was cooled, and the solid formed was filtered, dried and crystallized from acetic acid.

### 8-Bromo-2-(2-chlorophenyl)-7,9-dimethyl-2,3-dihydropyrido[3',2':4,5]thieno[3,2-*d*]pyrimidin-4(1*H*)-one (7a)

Yield: 78%; mp: >300 °C; IR (cm^−1^): 3433, 3275 (NH), 2947, 2897 (CH-aliphatic), 1643 (C = O); ^1^H NMR (DMSO-*d*_6_) δ ppm 2.67 (s, 3H, CH_3_), 2.77 (s, 3H, CH_3_), 6.11–6.14 (dd, 1H, CH-2, *J*= 7.2 Hz, *J*= 3.2 Hz), 7.18 (d, 1H, NH, *J*= 7.2 Hz, D_2_O exchangeable), 7.36–7.54 (m, 4H, Ar-H), 8.34 (d, 1H, NH, *J*= 3.2 Hz, D_2_O exchangeable); ^13^C NMR (DMSO-*d*_6_) δ ppm 19.7, 26.7 (CH_3_), 64.3 (CH-2), 110.5, 121.4, 124.5, 127.4, 128.5, 130.5, 132.4, 132.4, 137.8, 144.2, 144.7, 157.4, 159.5 (aromatic carbons), 161.8 (C = O); Anal. Calcd for C_17_H_13_BrClN_3_OS: C, 48.30; H, 3.10; N, 9.94. Found: C, 48.53; H, 3.07; N, 10.19.

### 8-Bromo-2-(2-fluorophenyl)-7,9-dimethyl-2,3-dihydropyrido[3',2':4,5]thieno[3,2-*d*]pyrimidin-4(1*H*)-one (7b)

Yield: 78%; mp: 292–293 °C; IR (cm^−1^): 3410, 3275 (NH), 2954, 2908 (CH-aliphatic), 1635 (C = O); ^1^H NMR (DMSO-*d*_6_) δ ppm 2.66 (s, 3H, CH_3_), 2.76 (s, 3H, CH_3_), 6.13–6.16 (dd, 1H, CH-2, *J*= 6.4 Hz, *J*= 2.8 Hz), 7.11 (d, 1H, NH, *J*= 6.8 Hz, D_2_O exchangeable), 7.17–7.48 (m, 4H, Ar-H), 8.36 (d, 1H, NH, *J*= 2.8 Hz, D_2_O exchangeable); ^13^C NMR (DMSO-*d*_6_) δ ppm 19.8, 26.7 (CH_3_), 61.8 (CH-2), 110.4, 116.1, 116.3, 121.4, 124.5, 128.0, 128.5, 130.9, 144.4, 144.7, 157.3, 159.1, 159.5 (aromatic carbons), 161.7 (C = O); Anal. Calcd for C_17_H_13_BrFN_3_OS: C, 50.26; H, 3.23; N, 10.34. Found: C, 50.49; H, 3.29; N, 10.50.

### 8-Bromo-2-(2-hydroxyphenyl)-7,9-dimethyl-2,3-dihydropyrido[3',2':4,5]thieno[3,2-*d*]pyrimidin-4(1*H*)-one (7c)

Yield: 74%; mp: >300 °C; IR (cm^−1^): 3421, 3390, 3309 (NH/OH), 2924, 2854 (CH-aliphatic), 1662 (C = O); ^1^H NMR (DMSO-*d*_6_) δ ppm 2.62 (s, 3H, CH_3_), 2.73 (s, 3H, CH_3_), 6.06–6.08 (dd, 1H, CH-2, *J*= 6.0 Hz, *J*= 2.8 Hz), 6.77 (d, 1H, NH, *J*= 6.4 Hz, D_2_O exchangeable), 6.80–7.41 (m, 4H, Ar-H), 7.99 (d, 1H, NH, *J*= 2.8 Hz, D_2_O exchangeable), 9.99 (s, 1H, OH, D_2_O exchangeable); MS m/z: 403 [(M + 2-H_2_)^+^, 19.99%], 401 [(M-H_2_)^+^, 16.19%], 93[C_6_H_4_OH^+^, 25.25%], 91 [100%]; Anal. Calcd for C_17_H_14_BrN_3_O_2_S: C, 50.50; H, 3.49; N, 10.39. Found: C, 50.78; H, 3.55; N, 10.56.

### 8-Bromo-7,9-dimethyl-2-[2-(trifluoromethyl)phenyl]-2,3-dihydropyrido[3',2':4,5]thieno[3,2-*d*]pyrimidin-4(1*H*)-one (7d)

Yield: 31%; mp: 267–268 °C; IR (cm^−1^): 3394, 3286 (NH), 2974, 2920 (CH-aliphatic), 1658 (C = O); ^1^H NMR (DMSO-*d*_6_) δ ppm 2.66 (s, 3H, CH_3_), 2.71 (s, 3H, CH_3_), 6.16 (d, 1H, CH-2, *J*= 6.4 Hz), 6.87 (d, 1H, NH, *J*= 7.2 Hz, D_2_O exchangeable), 7.60–7.94 (m, 4H, Ar-H), 8.37 (s, 1H, NH, D_2_O exchangeable); MS m/z: 457 [(M + 2)^+^, 10.14%], 455 [M^+^, 17.43%], 403 [100%]; Anal. Calcd for C_18_H_13_BrF_3_N_3_OS: C, 47.38; H, 2.87; N, 9.21. Found: C, 47.52; H, 2.91; N, 9.44.

### General procedure for the synthesis of 8-bromo-7,9-dimethyl-2-alkyl-pyrido[3',2':4,5]thieno[3,2-*d*]pyrimidin-4(3*H*)-ones 8a–c

A mixture of 3-aminothieno[2,3-*b*]pyridine-2-carboxamide **4** (0.6 g, 2 mmol) and the appropriate acid anhydride (5 mL) was heated under reflux for 5 h. The reaction was cooled, poured onto ice-cold water (50 mL) and left overnight. The solid formed was filtered, dried and crystallized from the suitable solvent.

### 8-Bromo-2,7,9-trimethylpyrido[3',2':4,5]thieno[3,2–*d*]pyrimidin-4(3*H*)-one (8a)

Crystallized from acetic acid. Yield: 72%; mp: >300 °C; IR (cm^−1^): 3417 (NH), 2924, 2870 (CH-aliphatic), 1670 (C = O); ^1^H NMR (DMSO-*d*_6_) δ ppm 2.46 (s, 3H, CH_3_), 2.72 (s, 3H, CH_3_), 3.01 (s, 3H, CH_3_), 12.95 (s, 1H, NH, D_2_O exchangeable); MS m/z: 325 [(M + 2)^+^, 94.00%], 323 [M^+^, 100%]; Anal. Calcd for C_12_H_10_BrN_3_OS: C, 44.46; H, 3.11; N, 12.96. Found: C, 44.70; H, 3.17; N, 13.15.

### 8-Bromo-7,9-dimethyl-2-(trifluoromethyl)pyrido[3',2':4,5]thieno[3,2-*d*]pyrimidin-4(3*H*)-one (8b)

Crystallized from acetic acid. Yield: 61%; mp: 253–254 °C; IR (cm^−1^): 3197 (NH), 2924, 2900 (CH-aliphatic), 1662 (C = O); ^1^H NMR (DMSO-*d*_6_) δ ppm 2.78 (s, 3H, CH_3_), 3.06 (s, 3H, CH_3_), 12.88 (s, 1H, NH, D_2_O exchangeable); ^13^C NMR (DMSO-*d*_6_) δ ppm 18.9, 26.6 (CH_3_), 117.9, 120.7, 121.0, 123.0, 124.6, 146.7, 159.3, 159.9, 162.2 (CF_3_ and aromatic carbons), 172.4 (C = O); MS m/z: 379 [(M + 2)^+^, 7.71%], 377 [M^+^, 3.94%], 42 [100%]; Anal. Calcd for C_12_H_7_BrF_3_N_3_OS: C, 38.11; H, 1.87; N, 11.11. Found: C, 38.24; H, 1.90; N, 11.23.

### 8-Bromo-2-ethyl-7,9-dimethylpyrido[3',2':4,5]thieno[3,2-*d*]pyrimidin-4(3*H*)-one (8c)

Crystallized from DMF. Yield: 73%; mp: >300 °C; IR (cm^−1^): 3421 (NH), 2981, 2920 (CH-aliphatic), 1670 (C = O); ^1^H NMR (DMSO-*d*_6_) δ ppm 1.26 (t, 3H, CH_3_CH_2_, *J*= 7.6 Hz), 2.71 (s, 3H, CH_3_), 2.71–2.75 (q, 2H, CH_3_CH_2_, *J*= 7.6 Hz), 2.95 (s, 3H, CH_3_), 12.78 (s, 1H, NH, D_2_O exchangeable); Anal. Calcd for C_13_H_12_BrN_3_OS: C, 46.16; H, 3.58; N, 12.42. Found: C, 46.42; H, 3.63; N, 12.66.

### 8-Bromo-7,9-dimethyl-2-(2-oxopropyl)pyrido[3',2':4,5]thieno[3,2-*d*]pyrimidin-4(3*H*)-one (9)

A mixture of 3-aminothieno[2,3-*b*]pyridine-2-carboxamide **4** (0.6 g, 2 mmol) and ethyl acetoacetate (6 mL) was heated under reflux for 8 h. The reaction was cooled and the solid formed was filtered, washed with ethanol (2 × 25 mL), dried and crystallized from acetic acid.

Yield: 42%; mp: 266–267 °C; IR (cm^−1^): 3410 (NH), 2924, 2819 (CH-aliphatic), 1685, 1639 (C = O); ^1^H NMR (DMSO-*d*_6_) δ ppm 2.28 (s, 3H, CH_3_), 2.67 (s, 3H, CH_3_), 2.93 (s, 3H, CH_3_), 3.96 (s, 2H, CH_2_), 12.84 (s, 1H, NH, D_2_O exchangeable); MS m/z: 367 [(M + 2)^+^, 6.95%], 365 [M^+^, 5.19%], 43 [CH_3_CO^+^, 100%]; Anal. Calcd for C_14_H_12_BrN_3_O_2_S: C, 45.91; H, 3.30; N, 11.47. Found: C, 46.09; H, 3.37; N, 11.59.

### Ethyl (8-bromo-7,9-dimethyl-4-oxo-3,4-dihydropyrido[3',2':4,5]thieno[3,2-*d*]pyrimidin-2-yl)acetate (10)

A mixture of 3-aminothieno[2,3-*b*]pyridine-2-carboxamide **4** (0.6 g, 2 mmol) and diethyl malonate (6 mL) was heated under reflux for 8 h. The reaction was cooled and the solid was filtered, washed with ethanol (2 × 25 mL), dried and crystallized from acetic acid.

Yield: 84%; mp: 277–278 °C; IR (cm^−1^): 3294 (NH), 2924, 2854 (CH-aliphatic), 1747, 1658 (C = O); ^1^H NMR (DMSO-*d*_6_) δ ppm 1.21 (t, 3H, CH_3_CH_2_, *J*= 7.2 Hz), 2.68 (s, 3H, CH_3_), 2.92 (s, 3H, CH_3_), 3.86 (s, 2H, CH_2_), 4.14–4.19 (q, 2H, CH_3_CH_2_, *J*= 7.2 Hz), 13.04 (s, 1H, NH, D_2_O exchangeable); MS m/z: 397 [(M + 2)^+^, 100%], 395 [M^+^, 93.05%], 352 [(M + 2- C_2_H_5_O)^+^, 4.65%], 350 [(M- C_2_H_5_O)^+^, 5.05%]; Anal. Calcd for C_15_H_14_BrN_3_O_3_S: C, 45.47; H, 3.56; N, 10.60. Found: C, 45.80; H, 3.61; N, 10.84.

### (8-Bromo-7,9-dimethyl-4-oxo-3,4-dihydropyrido[3',2':4,5]thieno[3,2-*d*]pyrimidin-2-yl)acetic acid (11)

A mixture of the acetate ester **10** (0.4 g, 1 mmol) and 10% alc. KOH solution (1 g KOH in 10 mL ethanol) was heated under reflux for 4 h. The reaction was cooled, diluted with water (25 mL) and acidified with conc. HCl. The precipitate was filtered, dried and crystallized from DMF.

Yield: 65%; mp: >300 °C; IR (cm^−1^): 3433, 3248 (NH/OH), 2924, 2854 (CH-aliphatic), 1720, 1666 (C = O); ^1^H NMR (CF_3_COOD, DMSO-*d*_6_) δ ppm 2.66 (s, 3H, CH_3_), 2.93 (s, 3H, CH_3_), 3.75 (s, 2H, CH_2_); Anal. Calcd for C_13_H_10_BrN_3_O_3_S: C, 42.41; H, 2.74; N, 11.41. Found: C, 42.69; H, 2.72; N, 11.56.

## Pim-1 kinase inhibitory activity

### Materials and methods

The kinase inhibitory activity of the synthesized compounds was determined using the Kinexus compound profiling service, Canada. All the compounds were tested for their inhibitory activity against pim-1 kinase at 50 μM. The kinase used was cloned, expressed and purified using proprietary methods. Quality control testing is routinely performed to ensure compliance to acceptable standards. ^33^P-ATP was purchased from PerkinElmer. All other materials were of standard laboratory grade.

### Pim-1 kinase protein assay

The protein kinase target profiling was executed via employing a radioisotope assay format. All the assays were performed in a prepared radioactive working area. The protein kinase profiling assays were performed at room temperature for 20–30 min in a final volume of 25 μL according to the following assay reaction components: Component 1; 5 μl of diluted active protein kinase (∼10–50 nM final concentration in the assay). Component 2; 5 μl of stock solution of substrate. Component 3; 5 μl of kinase assay buffer. Component 4; 5 μl of the test compound, Staurosporine at 1 μM or 10% DMSO. Component 5; 5 μl of ^33^P-ATP (250 μM stock solution, 0.8 μCi).

**Figure 4.**
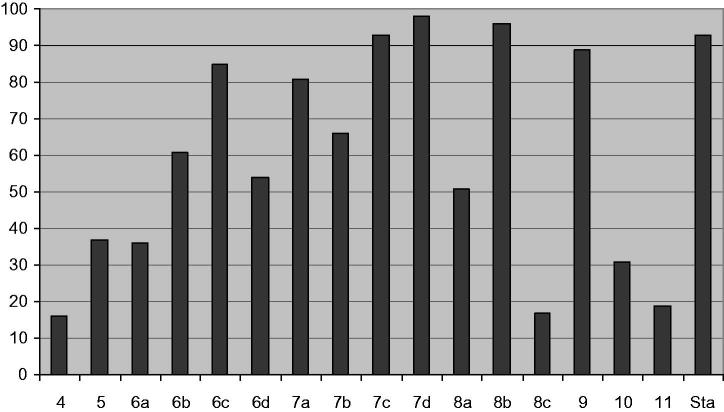
Percent inhibition of the test compounds and staurosporine (Sta) against pim-1 kinase.

The assay was initiated by the addition of ^33^P-ATP, followed by incubation at ambient temperature for 30 min. The assay was then terminated by spotting 10 μL of the reaction mixture onto a Multiscreen phosphocellulose P81 plate. The Multiscreen phosphocellulose P81 plate was washed three times for approximately 15 min each in a 1% phosphoric acid solution. The radioactivity on the P81 plate was counted in the presence of scintillation fluid in a Trilux scintillation counter. Blank control was set up that included all the assay components except the addition of the appropriate substrate (replaced with equal volume of assay dilution buffer). The corrected activity for protein kinase target was determined by removing the blank control value. The results were displayed in terms of percent inhibition and IC_50_ for the most active compounds. Table 1 and Figure 4 showed the obtained results.

**Table 1.** Results of pim-1 kinase inhibition achieved by the test compounds at 50 μM.


Compound no.	*R*	% Inhibition	**IC_**50**_ in μM**
**4**	–	16	ND^a^
**5**	H	37	ND
**6a**	2-ClC_6_H_4_	36	>100
**6b**	2-FC_6_H_4_	61	ND
**6c**	2-OHC_6_H_4_	**85**	4.62 ± 0.039
**6d**	2-CF_3_C_6_H_4_	54	ND
**7a**	2-ClC_6_H_4_	**81**	**1.18 ± 0.14**
**7b**	2-FC_6_H_4_	66	ND
**7c**	2-OHC_6_H_4_	**93**	**1.38 ± 0.025**
**7d**	2-CF_3_C_6_H_4_	**98**	**1.97 ± 0.022**
**8a**	CH_3_	51	ND
**8b**	CF_3_	**96**	8.83 ± 0.028
**8c**	C_2_H_5_	17	ND
**9**	CH_2_COCH_3_	**89**	4.18 ± 0.076
**10**	CH_2_COOC_2_H_5_	31	ND
**11**	CH_2_COOH	19	ND
**Staurosporine** (1μM)		93	ND

Bold values indicated the most potent compounds.

a ND: means not determined.

### *In vitro* cytotoxic activity

#### Cell culture

Cancer cells from different cancer cell lines were purchased from American type Cell Culture collection (ATCC, Manassas, VA). The cell lines used in this study were human breast adenocarcinoma (MCF7), human colon adenocarcinoma (HCT116) and human prostate cancer cells (PC3). The cell lines were grown on the appropriate growth medium Dulbecco's modified Eagle's medium (DMEM) or Roswell Park Memorial Institute medium (RPMI 1640) supplemented with 100 mg/mL of streptomycin, 100 units/mL of penicillin and 10% of heat-inactivated fetal bovine serum in a humidified, 5% (v/v) CO_2_ atmosphere at 37 °C.

#### Cytotoxicity assay by 3-[4,5-dimethylthiazole-2-yl]-2,5-diphenyltetrazolium bromide (MTT)

**Figure 5.**
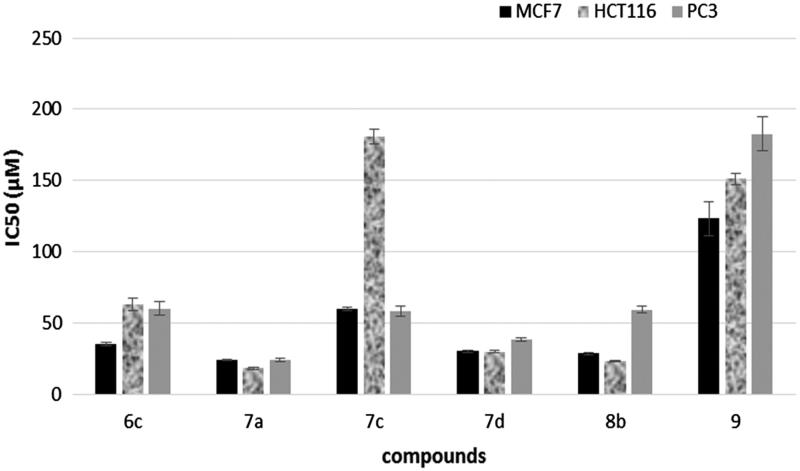
IC_50_ in μM of compounds **6c, 7a, 7c, 7d, 8b and 9** on three cell lines.

Exponentially growing cells from different cancer cell lines were trypsinized, counted and seeded at the appropriate densities (2000–10 000 cells/0.33 cm^2^ well) into 96-well microtiter plates. The cells were incubated in a humidified atmosphere at 37°C for 24 h. Then, the cells were exposed to different concentrations of compounds **6c**, **7a, 7c, 7d**, **8b** and **9** (0.1, 10, 100 and 1000 μM) for 72 h. The viability of the treated cells was determined using MTT technique. The media were removed; cells were incubated with 200 μL of 5% MTT solution/well (Sigma Aldrich, St. Louis, MO) and were allowed to metabolize the dye into colored-insoluble formazan crystals for 2 h. The remaining MTT solution was discarded from the wells and the formazan crystals were dissolved in 200 μL/well-acidified isopropanol for 30 min, covered with aluminum foil and with continuous shaking using a MaxQ 2000 plate shaker (Thermo Fisher Scientific Inc, MI) at room temperature. The absorbance was measured at 570 nm using a Stat FaxR 4200 plate reader (Awareness Technology, Inc., Palm City, FL). The cell viability were expressed as percentage of control and the concentration that induces 50% of maximum inhibition of cell proliferation (IC_50_) was determined for each compound using Graph Pad Prism version 5 software (Graph Pad software Inc, CA)^32^^,^^33^. The results are shown in Table 2 and represented graphically in Figure 5.

**Table 2.** Results of *in vitro* cytotoxic screening of compounds **6c, 7a, 7c, 7d, 8b** and **9** on three cell lines.

	IC_50_ in μM^a^		
Compound no.	MCF7	HCT116	PC3
**6c**	35.5 ± 1.30	63.3 ± 4.50	60.12 ± 4.65
**7a**	23.8 ± 0.40	18.26 ± 0.68	24.2 ± 0.91
**7c**	59.6 ± 1.20	180.69 ± 5.03	58.16 ± 3.48
**7d**	30.2 ± 0.80	30.03 ± 0.55	38.4 ± 1.07
**8b**	28.6 ± 1.00	23.48 ± 0.53	59.9 ± 2.48
**9**	123.2 ± 12.00	151.33 ± 4.04	182.69 ± 12.07

a The values given are means of three experiments.

## Results and discussion

### Chemistry

The synthesis of the target compounds was outlined in Schemes 1–3.

The synthesis of the starting compound **4** was accomplished *via* alkaline hydrolysis of 3-amino-5-bromo-4,6-dimethylthieno[2,3-*b*]pyridine-2-carbonitrile (**3**). The latter compound was prepared as reported by Madkour et al.^30^. The formation of compound **4** was confirmed by IR spectroscopy that showed the disappearance of the characteristic CN band and the appearance of C = O band at 1666 cm^−1^ and two NH_2_ forked bands at 3464–3267 cm^−1^. The ^1^H NMR spectrum of the carboxamide derivative **4** showed two exchangeable NH_2_ signals at δ 6.89 and δ 7.25 ppm. It is noteworthy that compound **4** was previously reported as unexpected product during acid catalyzed hydrolysis of *N*2,*N*2-di(5-methyl-2-furylmethyl)-3-amino-4,6-dimethylthieno[2,3-*b*]pyridine-2-carboxamide^31^.

Reacting the 3-aminothieno[2,3-*b*]pyridine-2-carboxamide **4** with 2-substituted benzaldehydes in DMF and few drops of conc. HCl afforded 8-bromo-2-(2-substituted phenyl)-7,9-dimethylpyrido[3',2':4,5]thieno[3,2-*d*]pyrimidin-4(3*H*)-ones **6a**–**d**. The IR spectra of compounds **6a**–**d** showed one NH band at 3464–3309 cm^−1^ together with C = O band at 1670–1662 cm^−1^. Their ^1^H NMR spectra revealed the presence of one exchangeable singlet signal at δ 12.82–13.45 ppm corresponding to NH proton as well as the aromatic signals at δ 7–8 ppm. The ^13^C NMR spectra of **6a**, **6b** and **6d** showed a signal at δ 161–170 ppm corresponding to C = O carbon.

On the other hand, reacting compound **4** with 2-substituted benzaldehydes in acetic acid gave 2,3-dihydropyridothienopyrimidin-4-ones **7a**–**d**. The formation of the dihydro products was confirmed by the spectral data. Thus, the IR spectra of compounds **7a**–**d** showed two NH bands at 3433–3275 cm^−1^ and one C = O band at 1662–1635 cm^−1^. A bright evidence was obtained from the ^1^H NMR spectra of compounds **7a**–**d** that revealed the presence of two exchangeable doublet signals at δ 6.77–7.18 ppm and δ 7.99–8.37 ppm corresponding to two NH protons as well as a doublet of doublets signal at δ 6.06–6.16 ppm corresponding to CH-2 proton. Moreover, the ^13^C NMR spectra of compounds **7a**,**b** showed a signal at δ 61.8–64.8 ppm corresponding to CH-2 carbon together with the signals of the introduced aromatic carbons and the C = O carbons.

Reacting the starting compound **4** with different acid anhydrides afforded 2-methyl, 2-trifluoromethyl and 2-ethylpyridothienopyrimidinones **8a**–**c**. The IR spectroscopy confirmed the structures of **8a**–**c** through the appearance of NH band at 3421–3197 cm^−1^ and C = O band at 1662–1670 cm^−1^. Meanwhile, their ^1^H NMR spectra showed an NH exchangeable singlet signal at δ 12.78–12.95 ppm. The ^13^C NMR spectrum of compound **8b** showed a signal at δ 172.4 ppm corresponding to C = O carbon.

The reaction of the amino amide derivative **4** and ethyl acetoacetate or diethyl malonate gave the 2-(2-oxopropyl) derivative **9** and the acetate derivative **10**, respectively. The IR spectrum of both compounds showed two carbonyl bands at 1685 and 1639 cm^−1^ in case of compound **9** and 1747, 1658 cm^−1^ in case of compound **10**. The ^1^H NMR spectrum of compound **9** revealed the appearance of two singlet signals at δ 2.28 ppm and δ 3.96 ppm corresponding to the 2-oxopropyl protons as well as an exchangeable singlet signal at δ 12.84 ppm indicating the cyclization of the pyrimidine ring. Whilst, the ^1^H NMR spectrum of compound **10** showed a singlet signal at δ 3.86 ppm assigned to the methylene proton beside triplet and quartet signals assigned to the ethyl protons.

Alkaline hydrolysis of the ester group of compound **10** using 10% alc. KOH resulted in the formation of the acetic acid derivative **11**. Its IR spectrum indicated the absence of the ester C = O at 1747 cm^−1^ and the appearance of acidic C = O at 1720 cm^−1^. While, its ^1^H NMR spectrum revealed the absence of the characteristic triplet and quartet signals of the ester group.

### Pim-1 kinase inhibitory activity

All the compounds were tested for their ability to inhibit pim-1 kinase at 50 μM using the Kinexus compound profiling service, Canada and the results (in terms of percentage inhibition and IC_50_ for the active compounds) were displayed in Table 1 and represented graphically in Figure 4.

The results indicated that six compounds exhibited highly potent pim-1 inhibitory activity in the range of 81–98%. These compounds were **6c** (85%), **7a** (81%), **7c** (93%), **7d** (98%), **8b** (96%) and **9** (89%). Consequently, their IC_50_ were determined as: **6c** (4.62 μM), **7a** (1.18 μM), **7c** (1.38 μM), **7d** (1.97 μM), **8b** (8.83 μM) and **9** (4.18 μM).

Besides, four compounds showed moderate inhibitory activity in the range of 51–66% [compounds **6b** (61%), **6d** (54%), **7b** (66%) and **8a** (51%)]. The rest of the synthesized compounds displayed poor enzyme inhibitory activity. These results were far better than those obtained with thieno[2,3-*b*]pyridine derivatives^24^.

SAR study of the pyrido[3',2':4,5]thieno[3,2-*d*]pyrimidin-4(1*H*)-ones as pim-1 inhibitors indicated the following points:

- The 2-unsubstituted derivative **5** showed poor enzyme inhibitory activity (37%).
- Regarding the 2-(2-substituted phenyl) series (**6a**–**d**), it was found that the 2-chlorophenyl derivative **6a** gave poor inhibition of pim-1 kinase (36%) with IC_50_ >100 μM. However, other members of the same series showed moderate to potent activity (54–85%). The 2-hydroxyphenyl derivative **6c** showed the highest pim-1 inhibitory activity in this series (85% inhibition of pim-1 kinase with IC_50_ 4.62 μM).
- The 2-(2-substituted phenyl)-2,3-dihydro series (**7a**–**d**) afforded the most potent pim-1 inhibitors in this study. Members belonging to this series showed pim-1 inhibition in the range of 66-98% with IC_50_ values in the range of 1.18-1.97 μM. The 2-chlorophenyl derivative **7a** showed the highest pim-1 kinase inhibition with IC_50_ of 1.18 μM (compare with **6a**). Thus, it seemed that aromaticity of the pyrimidine ring was not essential for pim-1 inhibition. Further study of the effect of substitution at *meta* or *para* positions of the phenyl ring is still needed
- The 2-alkyl derivatives **8a**–**c** exhibited great variability in their activities as pim-1 inhibitors. Thus, while the 2-methyl derivative **8a** showed moderate pim-1 inhibition (51%), its replacement with 2-triflouromethyl group in **8b** enhanced the activity significantly (96% inhibition and IC_50_ of 8.83 μM). On the other hand, increasing the chain length into 2-ethyl group (compound **8c**) reduced the enzyme inhibition greatly (23%).
- Regarding the carbonyl containing alkyl series **9**–**11**, it was found that the oxopropyl derivative **9** showed potent pim-1 inhibitory activity (89% with IC_50_ of 4.18 μM). Nevertheless, the ethyl acetate derivative **10** and its acid derivative **11** gave poor pim-1 inhibition.

### *In vitro* cytotoxic activity

The most active pim-1 inhibitors in this study work, namely, compounds **6c**, **7a, 7c**, **7d**, **8b** and **9** were screened for their cytotoxic activity against three cell lines using MTT method^32^^,^^33^. The cell lines examined were the human breast adenocarcinoma (MCF7), the human colon adenocarcinoma (HCT116) and the human prostate cancer cells (PC3). The results in terms of IC_50_ in μM are given in Table 2 and represented graphically in Figure 5.

From the results, it can be concluded that MCF7 and HCT116 cell lines were more sensitive to the action of the compounds than PC3 cell line.

Compounds **7a** [the 2-(2-chlorophenyl)-2,3-dihydro derivative] and **7d** [the 2-(2-(trifluoromethyl)-phenyl)-2,3-dihydro derivative] displayed the most potent cytotoxic effect on the three cell lines tested with IC_50_ values between 18 and 38 μM. These results were consistent with their high kinase IC_50_ values. Whilst, compound **8b** [the 2-(trifluoromethyl) derivative] showed potent cytotoxic activity on MCF7 and HCT116 cell lines and moderate cytotoxic effect on PC3 cell line.

Both compounds **6c** and **7c** exhibited moderate cytotoxic effect on all the cell lines tested, whilst compound **9** displayed weak cytotoxic activity on the three cell lines.

Again, the results obtained here were better than those obtained with thieno[2,3-*b*]pyridine derivatives.

## Conclusion

Structure rigidification via ring closure proved to be a successful strategy to improve the pim-1 inhibitory activity as well as the cytotoxic activity of thieno[2,3-*b*]pyridines. In the present work, four series of pyridothienopyrimidin-4-one derivatives were designed and prepared as pim-1 inhibitors. While only one thieno[2,3-*b*]pyridine derivative displayed potent pim-1 inhibition with IC_50_ of 12.71 μM, six pyridothienopyrimidin-4-ones (**6c**, **7a**, **7c**, **7d**, **8b** and **9**) showed highly potent pim-1 inhibitory activity with IC_50_ of 4.62, 1.18, 1.38, 1.97, 8.83 and 4.18 μM, respectively. SAR study of pyridothienopyrimidin-4-ones indicated that the 2-(2-substituted phenyl)-2,3-dihydro series **7a**–**d** afforded the most potent pim-1 inhibitors. The most active compounds were tested for their cytotoxic activity on three cell lines [MCF7, HCT116 and PC3]. Compounds **7a** [the 2-(2-chlorophenyl)-2,3-dihydro derivative] and **7d** [the 2-(2-(trifluoromethyl)-phenyl)-2,3-dihydro derivative] exhibited the most potent cytotoxic activity on the three cell lines tested. A significant improvement of the cytotoxic activity was also noticed relative to the precursors thieno[2,3-*b*]pyridine derivatives. The results of the cytotoxicity were in good agreement with the pim-1 IC_50_ values. Further work on pyridothienopyrimidin-4-ones is still needed to obtain more potent pim-1 inhibitors and to improve the physicochemical properties of these derivatives.
